# Genomic Analysis of *Escherichia coli* Longitudinally Isolated from Broiler Breeder Flocks after the Application of an Autogenous Vaccine

**DOI:** 10.3390/microorganisms10020377

**Published:** 2022-02-06

**Authors:** Liča Lozica, Kasper Rømer Villumsen, Ganwu Li, Xiao Hu, Maja Maurić Maljković, Željko Gottstein

**Affiliations:** 1 Department of Poultry Diseases with Clinic, Faculty of Veterinary Medicine, University of Zagreb, Heinzelova 55, 10000 Zagreb, Croatia; llozica@vef.unizg.hr; 2 Department of Veterinary and Animal Sciences, Faculty of Health and Medical Sciences, University of Copenhagen, Dyrlægevej 88, 1870 Copenhagen, Denmark; krv@sund.ku.dk; 3 State Key Laboratory of Veterinary Biotechnology, Harbin Veterinary Research Institute, Chinese Academy of Agricultural Sciences, Harbin 150069, China; liganwu@iastate.edu; 4 Department of Veterinary Diagnostic and Production Animal Medicine, College of Veterinary Medicine, Iowa State University, Ames, IA 50011, USA; xiaohu@iastate.edu; 5 Department of Animal Breeding and Livestock Production, Faculty of Veterinary Medicine, University of Zagreb, Heinzelova 55, 10000 Zagreb, Croatia; mmauric@vef.unizg.hr

**Keywords:** *Escherichia coli*, colibacillosis, poultry, autogenous vaccine, whole-genome sequencing, SNP, AMR

## Abstract

*Escherichia coli* is the main bacterial cause of major economic losses and animal welfare issues in poultry production. In this study, we investigate the effect of an autogenous vaccine on *E. coli* strains longitudinally isolated from broiler breeder flocks on two farms. In total, 115 *E. coli* isolates were sequenced using Illumina technologies, and compared based on a single-nucleotide polymorphism (SNP) analysis of the core-genome and antimicrobial resistance (AMR) genes they carried. The results showed that SNP-based phylogeny corresponds to a previous multilocus-sequence typing (MLST)-based phylogeny. Highly virulent sequence types (STs), including ST117-F, ST95-B2, ST131-B2 and ST390-B2, showed a higher level of homogeneity. On the other hand, less frequent STs, such as ST1485, ST3232, ST7013 and ST8573, were phylogenetically more distant and carried a higher number of antimicrobial resistance genes in most cases. In total, 25 antimicrobial genes were detected, of which the most prevalent were *mdf*(*A*) (100%), *sitABCD* (71.3%) and *tet*(*A*) (13.91%). The frequency of AMR genes showed a decreasing trend over time in both farms. The highest prevalence was detected in strains belonging to the B1 phylogenetic group, confirming the previous notion that commensal strains act as reservoirs and carry more resistance genes than pathogenic strains that are mostly associated with virulence genes.

## 1. Introduction

*Escherichia coli (E. coli)* is the main bacterial cause of major economic losses and animal welfare issues in poultry production [1,2]. Colibacillosis on poultry farms is controlled through vaccination programs, in addition to strict biosecurity standards and rigorous farm management [1]. There are several available commercial *E. coli* vaccines, but no vaccination procedure to date has proved to be highly efficacious due to the genetic diversity of the bacteria [1]. As the avian pathogenic *E. coli* (APEC) is closely related to other extraintestinal pathogenic *E. coli* (ExPEC) subpathotypes, which cause infections in the human population, colibacillosis in poultry also presents a danger to public health [2,3,4,5,6,7,8].

The localization and severity of the infection varies depending on the strain, site of entry and general health of the bird [1,9]. Usually, the most common lesions in adult birds are airsacculitis, peritonitis, salpingitis and septicaemia, although in egg laying hens, peritonitis, salpingitis and salpingitis-peritonitis syndrome (SPS) have been considered the most prevalent, as the infection usually occurs by bacteria ascending through the cloaca [2,9,10,11].

Many studies have focused on the importance of certain virulence-associated genes (VAGs) and their role in the pathogenesis of *E. coli* infection [12,13,14,15]. The validation of specific sets of VAGs as definite predictors of *E. coli* virulence could help to improve the diagnostics process and enable quicker response during colibacillosis outbreaks. Currently, ColV plasmids are associated with the pathogenicity of *E. coli* and considered a defining trait of APEC [15,16,17]. However, Mageiros et al. (2021) have reported a high prevalence of the putative plasmid genes among both pathogenic and commensal *E. coli* strains in chickens, with a higher average number of plasmid genes per isolate in the commensal strains, suggesting that the virulence of *E. coli* is linked with the homologous sequence variations of the genes [18]. Several population genomics studies have detected signatures of adaptation to different hosts in the bacterial genomes, which are manifested as mutations or horizontal acquisition of genetic elements [8,19,20]. Additionally, the antimicrobial resistance (AMR) genes are often located on plasmids [21]. They are similarly easily acquired by horizontal transmission, which presents an additional growing concern for animal and human health [22].

Single-nucleotide polymorphisms (SNPs) are the most common form of genetic code variation in the genome [23]. They are considered the most useful biomarkers for disease diagnosis and prognosis because they can influence the rate of the disease progression and immune response of the host [23]. They can also be used to track transmission, predict important phenotypes of the bacteria and monitor disease outbreaks [24]. The aim of this study is to investigate the heterogeneity of the isolates using SNP analysis and to detect the prevalence and horizontal transmission of AMR genes between and within flocks on two broiler breeder farms after the application of the autogenous vaccine.

## 2. Material and Methods

### 2.1. Study Design

This study is a continuation of previous research on *E. coli* gene variability after the application of the autogenous vaccine [25]. While the previous study was focused on the frequency of virulence-associated genes and MLST, the focus of the present study was on the use of SNPs for the phylogenetic analysis and the effect of the autogenous vaccine on antimicrobial resistance gene prevalence.

Two broiler breeder farms that are part of the same company, Farm A and Farm B, with four and five flocks, respectively, were selected for the longitudinal research on *E. coli* gene variability after the application of the autogenous vaccine. The selected farms reported continuous problems with colibacillosis despite the regular use of commercial vaccines. From our suggestion, they started using an autogenous *E. coli* vaccine instead of the commercial vaccine, which proved to be successful [26]. Each flock was vaccinated with a specifically designed vaccine prepared from the strains isolated in the previous flock. In total, 115 *E. coli* strains originating from the flocks vaccinated with the commercial and/or autogenous vaccines were selected for whole-genome sequencing and further analyses (Table 1).

### 2.2. Bacterial Strain Selection and DNA Isolation

Bacterial strain selection, DNA isolation and sequencing were previously described in more detail [25]. Briefly, 115 *E. coli* strains were isolated from the daily mortalities diagnosed with colibacillosis. The carcasses were routinely subjected to the pathomorphological examination as a part of the health surveillance or during outbreaks. The strains were recovered from the birds with lesions consistent with colibacillosis and selected for whole-genome sequencing based on the tissue of origin and age of the birds. Target organs were the peritoneum, liver, oviduct, and bone marrow, as they are often severely affected by colibacillosis. In case the strains from targeted tissues were not available, strains from other organs, such as lungs and the pericardium, were selected. The strains originated from birds older than 21 weeks, when outbreaks caused by colibacillosis usually emerge. Three or more strains per flock used for the vaccine production were also sequenced and included in the analyses. DNA was isolated as previously described [25] and stored at −20 °C until further analyses. The isolates used in this study are described in Supplementary Table S1.

### 2.3. DNA Sequencing and Deposition

Sequencing libraries were created based on the Illumina technologies, following the manufacturer’s recommendations. Afterwards, whole-genome sequencing was conducted using MiSeq (Illumina, San Diego, CA, USA) with paired-end 250 bp for 40 isolates, and NovaSeq 6000 platform (Novogene Co., Ltd., Beijing, China) with paired-end 150 bp strategy for the remaining 75 isolates. The whole-genome sequences analyzed in this study were deposited in the National Center for Biotechnology Information (NCBI) as a BioProject under the accession number PRJNA681385.

### 2.4. Phylogenetic Analysis

SPAdes (v3.13.1) was used to assemble the raw reads of the 115 *E. coli* isolates to generate 115 draft genomes. Parsnp (v1.2) was used to construct the SNP tree, including 115 draft genomes and reference genomes, which were chosen based on their genetic diversity. In total, 19 reference genomes belonging to different phylogenetic groups and STs were included in this analysis [27]. All other genomes were aligned to the genome of the isolate B4, one of the input 115 sequences, to detect core SNPs and generate the core-genome SNP tree [28] by using Parsnp (Supplementary Table S2).

### 2.5. Antimicrobial Resistance Gene Analysis

For the analysis of the AMR genes, raw reads were assembled and trimmed using Assembler v1.2. [29]. Acquired antimicrobial resistance genes were detected using ResFinder v4.1. [30], and the assembled genomes were analysed with 90% ID and 60% minimum length threshold.

### 2.6. Statistical Analysis

The statistical analyses were performed in Statistica 13.2.0.17. (TIBCO Software Inc., Tulsa, OK, USA) and R 4.0.5. (R Core Team, Vienna, Austria). The results were tested for normality of data distribution using the Kolmogorov–Smirnov test. The statistical significance of differences in the frequency of AMR genes between and within both farms was analysed using the Kruskal–Wallis test, with statistical significance set at level *p* ≤ 0.05. Poisson regression analysis was used to calculate the incident rate, standard error (SE) and 95% confidence intervals (95% CI) between the antimicrobial treatment and AMR gene prevalence among different flocks.

## 3. Results and Discussion

**Figure 1:**
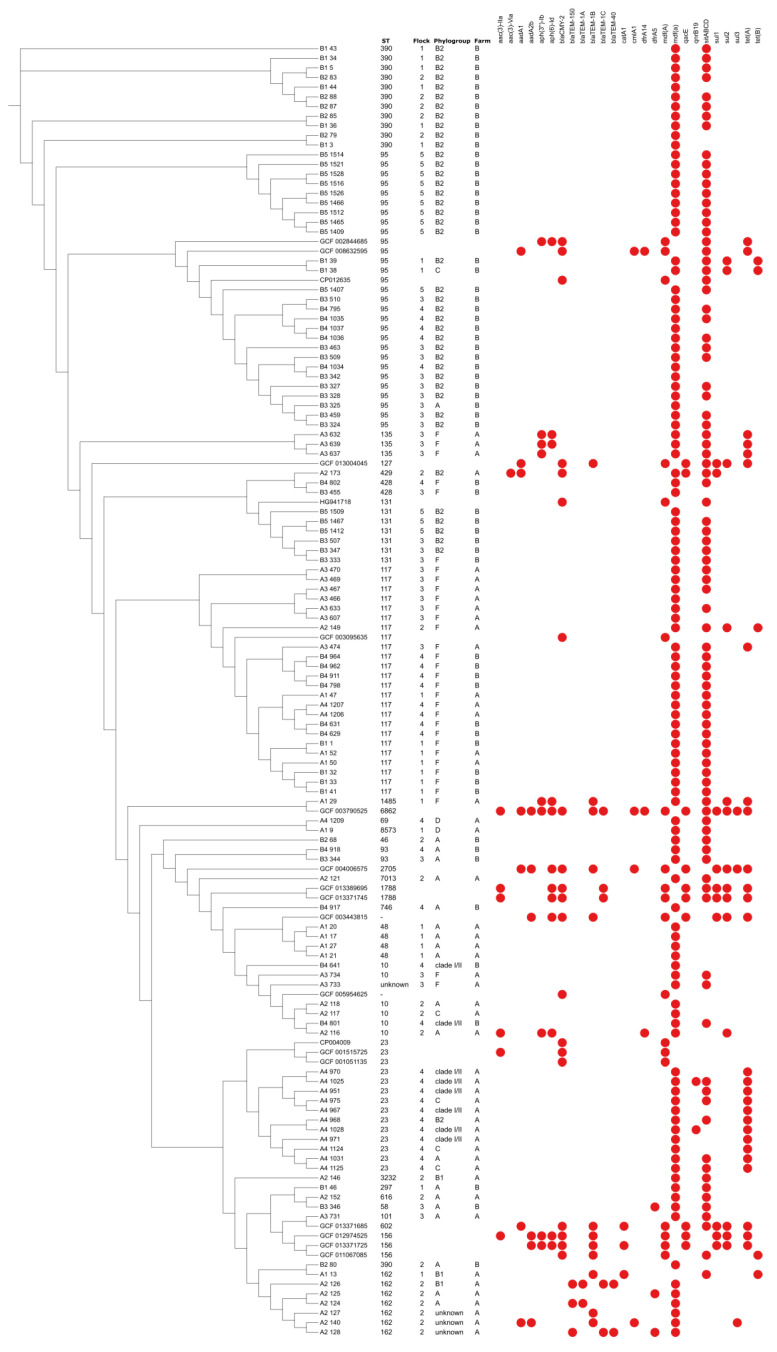
SNP-based phylogenetic tree showing distribution of AMR genes among 115 *E. coli* sequences. The analysis includes 19 reference *E. coli* genomes representing different STs and phylogenetic groups.

The results of the phylogenetic analysis correspond to the MLST-based phylogeny from the previous research (Figure 1) [25]. Isolates belonging to identical STs and phylogenetic groups were distributed both within the same and adjacent phylogroups in the phylogenetic tree based on the core-genome SNP clustering. Highly pathogenic strains, including ST117-F, ST95-B2, ST131-B2, and ST390-B2, formed larger clusters and showed higher homogeneity between the isolates originating from the same farm. This indicates their possible resistance to vaccination, despite continuous inclusion in the vaccine, and longitudinal spread to the consecutive flocks, but with reduced general clinical implications and improved production traits on Farm B. Additionally, ST117-F clustered together regardless of the flock or farm of origin, which has already been observed and explained by the mutual origin of the strains from the grandparent flocks or from the shared rearing houses [25,31]. ST162 and ST23 formed separate clusters and were detected only on Farm A. Both STs included a variety of phylogenetic groups of which most are considered commensal, although they have previously been isolated from both diseased and healthy poultry [32,33,34]. Vaccination on Farm A significantly influenced the selection of strains in the consecutive flocks, but also showed to be non-protective against the heterologous strains. As the majority of the strains on Farm A were low pathogenic, they were probably more easily controlled by vaccination compared to Farm B, where the majority of the strains were more pathogenic and the phylogroup B2 resisted vaccination. SNP genotyping generally reveals a more detailed clonal relationship between the investigated isolates [9]. In our study, it showed slightly more discriminatory results compared to the MLST-based phylogeny, showing distribution of several isolates with the same ST in adjacent clusters based on the core SNPs. The strains belonging to less frequent STs, such as ST1485, ST3232, ST7013 and ST8573, were phylogenetically more distant and usually clustered separately. However, their average number of detected AMR genes was variable, mostly depending on the phylogenetic group.

We identified 25 AMR genes, of which *mdf(A)* and *sitABCD* showed the highest prevalence on both farms (Table 2). Although *mdf(A)* has a broad-spectrum specificity, which includes six classes of antimicrobials, the phenotypes and the level of resistance it provides is unclear. Previous studies reported that *mdf(A)* encodes for a multidrug efflux pump and its expression confers multidrug resistance in *E. coli,* indicating that resistance could have occurred in isolates where we did not detect corresponding resistance genes [35,36]. Similar results were reported by Rafique et al. (2020), who detected the *mdf(A)* gene in all 92 investigated *E. coli* strains isolated from chickens in different Pakistani provinces [22].

The *SitABCD* system mediates the transport of iron and manganese. Its ability to obtain manganese contributes to the resistance to oxidative stress and protection against agents such as hydrogen peroxide [37]. Additionally, *sit* operon genes are often associated with clinical infections caused by ExPEC, and have a contributing role as virulence factors by mediating the metal ion transport [37,38]. ResFinder analyses of our isolates discovered multiple copies of *sitABCD* genes. Previous phylogenetic analyses of the *sit* operon originating from *E. coli* and *Shigella flexneri* revealed they are most likely acquired by several distinct genetic events involving horizontal gene transfer [38]. Said events probably contributed to the presence of multiple gene copies in a single isolate, from which some are plasmid borne, and some are carried on chromosomes, and led to their high occurrence in *E. coli* populations [16,38].

The gene *tet(A)* showed relatively high prevalence (13.91%), although it was detected only on Farm A and predominantly in Flock 4 (68.75%). The prevalence was probably a result of doxycycline application, which is commonly used as treatment for various bacterial poultry diseases. The dominance of *tet(A)* out of different tetracycline resistance genes was reported in *E. coli* isolated from chickens and several other animal species, as opposed to the strains isolated from humans where *tet(B)* was more prevalent [39,40,41,42].

Interestingly, mobilized colistin resistance (*mcr*) gene was not detected, although colistin (polymyxin E) has been used as antibiotic treatment on the studied farms (Table 1). It has been widely used to treat colibacillosis and as a growth promoter in the poultry industry for a long time [1,43,44]. Since polymyxins have been reintroduced as treatment for infections in humans and their application can influence the antimicrobial selective pressure [45], its use in veterinary medicine has been minimized [44].

**Figure 2:**
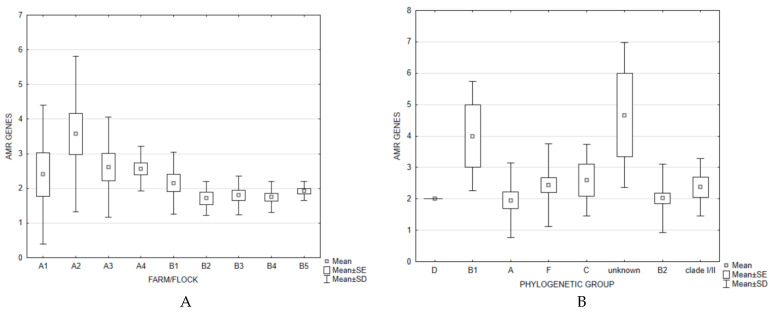
AMR gene frequency per flock on each farm (**A**) and per each detected phylogenetic group (**B**).

The frequency of AMR genes decreased over time on both farms, with a more apparent change on Farm A (Figure 2A). When considering the phylogenetic groups of the analysed isolates, the results showed the highest prevalence of AMR genes in the B1 phylogroup and strains belonging to untypeable phylogenetic groups (Figure 2B). The high prevalence of resistance genes in phylogroup B1 confirms the notion that commensal strains act as reservoirs of AMR genes. Previous studies indicated that commensal strains carry more AMR genes, as opposed to pathogenic strains that usually possess more virulence factors, but are more susceptible to antimicrobials [46,47]. This concept was confirmed by this study by the significantly higher frequency of AMR genes on the Farm A (Table 2), in addition to our previous study, which showed a significantly lower average frequency of VAGs on Farm A than on Farm B [25]. The strains of unknown phylogenetic groups were identified as ST162 (Supplementary Table S1). As other detected ST162 strains were identified as the A or B1 phylogenetic group, we assume that untypeable strains were variations of said commensal phylogroups, further confirming the highest prevalence of AMR genes in the commensal strains. Overall, the average number of AMR genes per isolate was 2.71 and 1.87 on Farm A and B, respectively, and there was no statistical significance in the prevalence of resistance genes between and within different studied flocks. The antimicrobial susceptibility testing of strains on the Farm A and B showed continuous decrease in resistance, especially on Farm A (unpublished data), which is consistent with the AMR gene results.

The results showed the treatment with antimicrobials possibly affected the prevalence of certain *E. coli* strains, in addition to the effect of autogenous vaccine application [48], although there was no correlation between the antimicrobials used in the flocks and the detected AMR genes (Supplementary Table S3). The implementation of the autogenous vaccine induced genetic homogenization of the isolates [26,48], which increased the selection pressure and alleviated the management of colibacillosis on the investigated farms. The present results correspond to previous studies, which reported that the application of autogenous vaccines alone or in combination with a commercial vaccine serves well as a preventive measure on poultry farms [49,50,51]. The results showed a probable positive interaction of the autogenous vaccine and antimicrobial treatment, which could have reduced the overall prevalence of AMR genes on the farms in the long term. The antimicrobials and the vaccine possibly interacted and formed a more powerful selection mechanism where strains with AMR genes, if not removed by treatment, could be controlled by the immune system. In addition, on the investigated farms, vaccination with autogenous vaccines reduced the need for treatment with antimicrobials, therefore the stimulation and emergence of resistant *E. coli* strains significantly decreased.

## 4. Conclusions

Genomic analysis showed that both MLST and SNP-based phylogeny can provide a detailed characterization of *E. coli* strains, taking into consideration their virulence and overall genetic relatedness. Although none of the STs or phylogenetic groups could be associated with specific AMR genes, strains belonging to more uncommon STs and commensal phylogenetic groups carried more AMR genes in most cases. The results showed the highest prevalence of *mdf(A)* and *sit* operon genes, which provide a wide-spectrum protection, indicating that the bacterial population was adapted over time by carrying less specialized genes. The autogenous vaccine induced the lower heterogeneity of the strains and possibly interacted with the antimicrobial treatment leading to the selection of strains with a lower amount of AMR genes over time, which is consistent with previous research.

## Figures and Tables

**Table 1:** Description of *E. coli* vaccination programs and antimicrobial treatment data (rearing and production period) for each flock in this study ^1^.

Farm	Flock	Number of Analysed Strains per Flock	Vaccination	Age at the Time of Vaccination	Treatment
**A**	1	10	Commercial vaccines (live attenuated + inactivated 2x)	0 d 10 w 18 w	Doxycycline
	2	14	Autogenous vaccine 2x	10 w 18 w	Doxycycline, tiamulin
	3	13	Autogenous vaccine 2x	12 w 19 w	-
	4	14	Autogenous vaccine 2x	10 w 19 w	Amoxicillin, doxycycline, enrofloxacin, oxytetracycline
**B**	1	13	Commercial vaccines (live attenuated + inactivated 2x)	0 d 13 w 19 w	Doxycycline, enrofloxacin, tylosin
	2	7	Commercial vaccines (live attenuated 2x) Autogenous vaccine 2x	0 d 5 w 11 w 18 w	Amoxicilllin, polymyxin E, tylosin
	3	15	Autogenous vaccine 2x	9 w 20 w	Doxycycline, polymyxin E
	4	16	Autogenous vaccine 2x	10 w 18 w	Doxycycline, enrofloxacin
	5	13	Autogenous vaccine 2x	9 w 17 w	Amoxicillin, enrofloxacin

^1^ adapted from Lozica et al., 2021.

**Table 2:** Prevalence of the identified acquired AMR genes (*n* (%)).

AMR Gene	Phenotype	Number of AMR Genes on Farm A * *n* = 51	Number of AMR Genes on Farm B ** *n* = 64	Total Number of AMR Genes
*aac(3)-IIa*	apramycin, gentamicin, tobramycin, dibekacin, netilmicin, sisomicin	1 (1.96)	0	1 (0.87)
*aac(3)-VIa*	gentamicin	1 (1.96)	0	1 (0.87)
*aadA1*	spectinomycin	2 (3.92)	0	2 (1.74)
*aadA2b*	spectinomycin, streptomycin	1 (1.96)	0	1 (0.87)
*aph(3″)-Ib*	streptomycin	5 (9.8)	0	5 (4.35)
*aph(6)-Id*	streptomycin	5 (9.81)	0	5 (4.35)
*bla*_CMY-2_	amoxicillin, amoxicillin + clavulanic acid, ampicillin, ampicillin + clavulanic acid, cefotaxime, cefotxitin, ceftazidime, piperacillin, piperacillin + tazobactam, ticarcillin, ticarcillin + clavulanic acid	1 (1.96)	0	1 (0.87)
*bla*_TEM-1A_	amoxicillin, ampicillin, cephalothin, piperacillin, ticarcillin	3 (5.88)	0	3 (2.61)
*bla*_TEM-1B_	amoxicillin, ampicillin, cephalothin, piperacillin, ticarcillin	4 (7.84)	0	4 (3.48)
*bla*_TEM-1C_	amoxicillin, ampicillin, cephalothin, piperacillin, ticarcillin	2 (3.92)	0	2 (1.74)
*bla*_TEM-40_	amoxicillin, amoxicillin + clavulanic acid, ampicillin, ampicillin + clavulanic acid, piperacillin, piperacillin + tazobactam, ticarcillin, ticarcillin + clavulanic acid	2 (3.92)	0	2 (1.74)
*bla*_TEM-150_	unknown beta-lactam	3 (5.88)	0	3 (2.61)
*catA1*	chloramphenicol	1 (1.96)	0	1 (0.87)
*cmIA1*	chloramphenicol	1 (1.96)	0	1 (0.87)
*dfrA5*	trimethoprim	2 (3.92)	1 (1.56)	3 (2.61)
*dfrA14*	trimethoprim	1 (1.96)	0	1 (0.87)
*mdf(A)*	unknown macrolide, aminoglycoside, tetracycline, fluoroquinolone, phenicol and rifamycin	51 (100)	64 (100)	115 (100)
*qacE*	benzylkonium chloride, ethidium bromide, chlorhexidine, cetylpyridinium	1 (1.96)	0	1 (0.87)
*qnrB19*	ciprofloxacin	2 (3.92)	0	2 (1.74)
*sitABCD*	hydrogen peroxide	31 (60.78)	51 (79.69)	82 (71.3)
*sul1*	sulfamethoxazole	1 (1.96)	0	1 (0.87)
*sul2*	sulfamethoxazole	3 (5.88)	2 (3.13)	5 (4.35)
*sul3*	sulfamethoxazole	1 (1.96)	0	1 (0.87)
*tet(A)*	doxycycline, tetracycline	17 (33.33)	0	17 (14.78)
*tet(B)*	doxycycline, tetracycline, minocycline	2 (3.92)	2 (3.13)	4 (3.48)

*, ** Statistically significant difference in AMR gene frequency was detected between the farms (*p* ≤ 0.01).

## Data Availability

The whole-genome sequences analysed in this study are publicly available as BioProject under the accession number PRJNA681385.

## Supplementary Materials

The following supporting information can be downloaded at: https://www.mdpi.com/article/10.3390/microorganisms10020377/s1, Table S1: Description of isolates used in this study. Isolates in bold were used for the production of autogenous vaccines for the next consecutive flock on the farm, Table S2: Description of reference genomes used for the SNP analysis, Table S3: The results of the Poisson regression analysis showing the incident rate, standard error (SE) and 95% confidence intervals (95% CI) between the antimicrobial treatment and AMR gene prevalence among different flocks.
