# Supplementary material for: Genomic Analysis of Escherichia coli Longitudinally Isolated from Broiler Breeder Flocks after the Application of an Autogenous Vaccine
Source: Microorganisms. 2022 Feb 6;10(2):377. doi: 10.3390/microorganisms10020377 (PMC8879504; doi:10.3390/microorganisms10020377)
Supplement: Supplementary file 1 [file microorganisms-10-00377-s001.zip › microorganisms-1521995-supplementary/Supplementary Table S1.pdf]

Supplementary Table S1. Description of isolates used in this study. Isolates in bold were used for the production of autogenous vaccines for the next consecutive flock on the farm.

Isolate	ST	Phylogroup	Accession number
**A1_9** [1]	8573	D	SAMN16954579
**A1_13**	162	B1	SAMN16954580
A1_17	48	A	SAMN16954581
A1_20	48	A	SAMN16954582
A1_21	48	A	SAMN16954583
A1_27	48	A	SAMN16954584
A1_29	1485	F	SAMN16954585
A1_47	117	F	SAMN16954586
**A1_50**	117	F	SAMN16954587
A1_52	117	F	SAMN16954588
A2_116	10	A	SAMN16954591
A2_117	10	C	SAMN16954592
**A2_118**	10	A	SAMN16954593
**A2_121**	7013	A	SAMN16954594
A2_124	162	A	SAMN16954595
**A2_125**	162	A	SAMN16954596
A2_126	162	B1	SAMN16954597
A2_127	162	unknown	SAMN16954598
A2_128	162	unknown	SAMN16954599
A2_140	162	unknown	SAMN16954600
A2_146	3232	B1	SAMN16954601
A2_149	117	F	SAMN16954602
A2_152	616	A	SAMN16954603
A2_173	429	B2	SAMN16954604
A3_466	117	F	SAMN16954605
**A3_467**	117	F	SAMN16954606
A3_469	117	F	SAMN16954607
A3_470	117	F	SAMN16954608
A3_474	117	F	SAMN16954609
**A3_607**	117	F	SAMN16954612
**A3_632**	135	F	SAMN16954613

A3_633	117	F	SAMN16954614
A3_637	135	F	SAMN16954615
**A3_639**	135	F	SAMN16954616
A3_731	101	A	SAMN16966892
A3_733	unknown	F	SAMN16966893
A3_734	10	F	SAMN16966894
A4_951	23	clade I/II	SAMN16966900
A4_967	23	clade I/II	SAMN16966902
A4_968	23	B2	SAMN16966903
A4_970	23	clade I/II	SAMN16967643
A4_971	23	clade I/II	SAMN16967644
A4_975	23	C	SAMN16967645
A4_1025	23	clade I/II	SAMN16967646
**A4_1028**	23	clade I/II	SAMN16967647
A4_1031	23	A	SAMN16967648
**A4_1124**	23	C	SAMN16967651
**A4_1125**	23	C	SAMN16967652
A4_1206	117	F	SAMN16967653
**A4_1207**	117	F	SAMN16967654
A4_1209	69	D	SAMN16967682
**B1_1**	117	F	SAMN16951411
**B1_3**	390	B2	SAMN16951412
**B1_5**	390	B2	SAMN16951413
**B1_32**	117	F	SAMN16951414
**B1_33**	117	F	SAMN16951415
B1_34	390	B2	SAMN16951416
B1_36	390	B2	SAMN16951417
B1_38	95	C	SAMN16951418
B1_39	95	B2	SAMN16951419
**B1_41**	117	F	SAMN16951420
B1_43	390	B2	SAMN16951421
**B1_44**	390	B2	SAMN16951422
**B1_46**	297	A	SAMN16951423
B2_68	46	A	SAMN16954590
**B2_79**	390	B2	SAMN16951424

**B2_80**	390	A	SAMN16951425
**B2_83**	390	B2	SAMN16951426
**B2_85**	390	B2	SAMN16951427
**B2_87**	390	B2	SAMN16951428
**B2_88**	390	B2	SAMN16951429
B3_324	95	B2	SAMN16951430
B3_325	95	A	SAMN16951431
B3_327	95	B2	SAMN16951432
B3_328	95	B2	SAMN16951433
B3_333	131	F	SAMN16951434
B3_342	95	B2	SAMN16951435
B3_344	93	A	SAMN16951436
B3_346	58	A	SAMN16951437
B3_347	131	B2	SAMN16951438
B3_455	428	F	SAMN16951439
B3_459	95	B2	SAMN16951440
B3_463	95	B2	SAMN16951441
**B3_507**	131	B2	SAMN16954610
**B3_509**	95	B2	SAMN16951442
**B3_510**	95	B2	SAMN16954611
B4_629	117	F	SAMN16951443
B4_631	117	F	SAMN16951444
B4_641	10	clade I/II	SAMN16951445
B4_795	95	B2	SAMN16966895
B4_798	117	F	SAMN16966896
B4_801	10	clade I/II	SAMN16966897
B4_802	428	F	SAMN16966898
B4_911	117	F	SAMN16951446
B4_917	746	A	SAMN16951447
B4_918	93	A	SAMN16966899
B4_962	117	F	SAMN16951448
**B4_964**	117	F	SAMN16966901
**B4_1034**	95	B2	SAMN16951449
B4_1035	95	B2	SAMN16967649
**B4_1036**	95	B2	SAMN16967650

B4_1037	95	B2	SAMN16951450
B5_1407	95	B2	SAMN16967683
B5_1409	95	B2	SAMN16967684
B5_1412	131	B2	SAMN16967685
B5_1465	95	B2	SAMN16967686
B5_1466	95	B2	SAMN16967687
B5_1467	131	B2	SAMN16967688
B5_1509	131	B2	SAMN16967689
B5_1512	95	B2	SAMN16976350
B5_1514	95	B2	SAMN16976351
B5_1516	95	B2	SAMN16976352
B5_1521	95	B2	SAMN16976353
B5_1526	95	B2	SAMN16976354
B5_1528	95	B2	SAMN16976355

[1] Labels of the isolates include farm, flock and isolate number (i.e. Farm A, Flock 1, isolate 9).
