# Supplementary material for: Genomic Analysis of Escherichia coli Longitudinally Isolated from Broiler Breeder Flocks after the Application of an Autogenous Vaccine
Source: Microorganisms. 2022 Feb 6;10(2):377. doi: 10.3390/microorganisms10020377 (PMC8879504; doi:10.3390/microorganisms10020377)
Supplement: Supplementary file 1 [file microorganisms-10-00377-s001.zip › microorganisms-1521995-supplementary/Supplementary Table S2.pdf]

Supplementary Table S2. Description of reference genomes used for the SNP analysis.

Isolate	Phylogenetic group	ST	Accession number
ECCNB20-2	A	ST2705	GCA_004006575.1
EK2009	A	unknown	GCA_005954625.1
E308	A	unknown	GCA_003443815.1
AH62	A	ST1788	GCA_013371745.1
AH65	A	ST1788	GCA_013389695.1
AH25	B1	ST156	GCA_013371725.1
C21	B1	ST156	GCA_012974525.1
3R	B1	ST156	GCA_011067085.1
AH01	B1	ST602	GCA_013371685.1
HB37	B2	ST127	GCA_013004045.1
ExPECXM	B2	ST95	GCA_002844685.1
ST95-32	B2	ST95	GCA_008632595.1
ACN001	C	ST23	GCA_001051135.1
ACN002	C	ST23	GCA_001515725.1
ECCNB12-2	F	ST6862	GCA_003790525.1
104	F	ST117	GCA_003095635.1
APEC O78	C	ST23	CP004009
SF-088	B2	ST95	CP012635
EC958	B2	ST131	HG941718
