# Supplementary material for: Genomic Analysis of Escherichia coli Longitudinally Isolated from Broiler Breeder Flocks after the Application of an Autogenous Vaccine
Source: Microorganisms. 2022 Feb 6;10(2):377. doi: 10.3390/microorganisms10020377 (PMC8879504; doi:10.3390/microorganisms10020377)
Supplement: Supplementary file 1 [file microorganisms-10-00377-s001.zip › microorganisms-1521995-supplementary/Supplementary Table S3.pdf]

Supplementary Table S3. The results of the Poisson regression analysis showing the incident rate, standard error (SE) and 95% confidence intervals (95% CI) between the antimicrobial treatment and AMR gene prevalence among different flocks. The first flock on both farms was used as a reference group for the analysis.

Farm	Flock	Incident rate	SE	2.5%	97.5%	p value
A	1	2.40	0.60	1.47	3.93	
	2	1.49	0.44	0.83	2.67	0.18
	3	1.09	0.32	0.62	1.93	0.77
	4	1.07	0.28	0.64	1.78	0.79
B	1	2.15	0.24	1.73	2.68	
	2	0.80	0.12	0.59	1.07	0.13
	3	0.84	0.11	0.64	1.09	0.19
	4	0.81	0.10	0.63	1.04	0.10
	5	0.89	0.11	0.71	1.12	0.34
